# Human Lung Mast Cell Products Regulate Airway Smooth Muscle CXCL10 Levels

**DOI:** 10.1155/2014/875105

**Published:** 2014-02-06

**Authors:** H. Alkhouri, V. Cha, K. Tong, L. M. Moir, C. L. Armour, J. M. Hughes

**Affiliations:** ^1^Faculty of Pharmacy, The University of Sydney, Sydney, NSW 2006, Australia; ^2^Discipline of Pharmacology and Woolcock Institute of Medical Research, The University of Sydney, Sydney, NSW 2006, Australia

## Abstract

In asthma, the airway smooth muscle (ASM) produces CXCL10 which may attract CXCR3^+^ mast/T cells to it. Our aim was to investigate the effects of mast cell products on ASM cell CXCL10 production. ASM cells from people with and without asthma were stimulated with IL-1**β**, TNF-**α**, and/or IFN**γ** and treated with histamine (1–100 **μ**M) ± chlorpheniramine (H1R antagonist; 1 **μ**M) or ranitidine (H2R antagonist; 50 **μ**M) or tryptase (1 nM) ± leupeptin (serine protease inhibitor; 50 **μ**M), heat-inactivated tryptase, or vehicle for 4 h or 24 h. Human lung mast cells (MC) were isolated and activated with IgE/anti-IgE and supernatants were collected after 2 h or 24 h. The supernatants were added to ASM cells for 48 h and ASM cell CXCL10 production detected using ELISA (protein) and real-time PCR (mRNA). Histamine reduced IL-1**β**/TNF-**α**-induced CXCL10 protein, but not mRNA, levels independent of H1 and H2 receptor activation, whereas tryptase and MC 2 h supernatants reduced all cytokine-induced CXCL10. Tryptase also reduced CXCL10 levels in a cell-free system. Leupeptin inhibited the effects of tryptase and MC 2 h supernatants. MC 24 h supernatants contained TNF-**α** and amplified IFN**γ**-induced ASM cell CXCL10 production. This is the first evidence that MC can regulate ASM cell CXCL10 production and its degradation. Thus MC may regulate airway myositis in asthma.

## 1. Introduction

In asthma, airway smooth muscle (ASM) cells may play a significant role in supporting inflammation locally. They produce a wide range of chemokines and growth factors *in vitro* in response to proinflammatory cytokines, which are elevated in asthma following release by activated inflammatory cells and other airway structural cells [1–3].

Degranulating mast cells (MC) are present in higher numbers in the ASM bundles of people with asthma than people without asthma [4–7]. MC numbers are further increased in allergic compared with nonallergic asthma [5] and thus may be a specific feature of eosinophilic asthma. There is conflicting evidence about the presence of MC in the ASM in severe asthma, which is often associated with neutrophilia and steroid resistance. Carroll et al. [4] found that degranulating MC numbers were highest in the ASM layer in fatal asthma, whereas Balzar et al. [8] found no evidence of them in the ASM in biopsies from a severe asthma cohort.

MC activation by allergen mediated cross-linking of IgE results in the release of a variety of preformed and newly synthesised mediators [9, 10]. These include the major granule-derived mediators histamine and tryptase and newly synthesised cysteinyl leukotrienes, whose effects on ASM contractility are well established. MC also produce other proteases, arachidonic acid metabolites, and a wide range of cytokines and growth factors [9, 10]. The relative balance of these mediators in the vicinity of the ASM cell will determine the overall effect MC have on ASM production of a certain chemokine. What role that chemokine plays in airway inflammation will depend on its quantity and activity locally.

To date, the effects of many MC products on ASM chemokine production are unknown. When studied individually, some MC mediators directly affect ASM chemokine production and thus may regulate inflammation locally. For example, interleukin (IL-)1*β* or tumour necrosis factor (TNF-)*α* alone [11] and IL-4 or IL-13 alone and in combination with IL-1*β* [12], induce ASM release of the potent eosinophil chemoattractant CCL11 (eotaxin), while we and others have shown that MC proteases cleave CCL11 [13, 14]. We have also shown that histamine enhances IL-1*β*-induced granulocyte-macrophage colony stimulating factor (GM-CSF) release but strongly inhibits CCL5 (RANTES) release by ASM cells from people with and without asthma [15]. Further studies are needed to investigate the pro- and anti-inflammatory effects of MC and whether or not they can regulate their own recruitment to the ASM and survival there.

Increased localization of MC to the ASM layer could be controlled by many ASM cell derived chemokines, as human lung MC (HLMC) express a range of chemokine receptors [16]. However MC in the ASM of people with asthma are CXCR3 positive, whereas less than half of the MC elsewhere in the airway submucosa express CXCR3. Further, ASM in biopsies from people with asthma are often (~50%) immunoreactive for the CXCR3 ligand CXCL10, whereas the ASM in biopsies from people without asthma are not [17]. *In vitro* we have established that asthmatic ASM cells produce CXCL10 more rapidly than nonasthmatic ASM cells under Th1 inflammatory conditions [18] and MC chemotaxis towards medium from the asthmatic ASM cells is driven by CXCL10 activating CXCR3 on the MC [17]. Whether or not MC can regulate ASM CXCL10 production and activity is not known.

Thus, the aims of this study were to investigate the effects of MC products on CXCL10 production by ASM cells from people with and without asthma. The effects of the granule-derived products histamine and tryptase, as well as the overall effects of human lung MC products released in the first 2 h or 2–24 h after activation, on ASM CXCL10 production were examined.

## 2. Methods

### 2.1. Agents

Recombinant Human IFN*γ* (BD Biosciences, Australia), IL-1*β*, and TNF*α* (R&D Systems, Minneapolis, MN) were reconstituted in sterile PBS containing 0.1% bovine serum albumin (BSA). Histamine, chlorpheniramine, and ranitidine (Sigma-Aldrich, Sydney, Australia) were reconstituted in water for irrigation (Baxter, Sydney, Australia). Human lung tryptase (≥5,000 mU/mg/mL of vehicle consisting of 1 M NaCl, 50 mM sodium acetate, 0.01% sodium azide, and 50 *μ*M heparin) was obtained from Calbiochem (La Jolla, CA, USA). BSA and leupeptin were purchased from Sigma-Aldrich. All agents were stored in aliquots at −20°C, except for IFN*γ* which was stored at −80°C.

### 2.2. Airway Smooth Muscle

ASM cell cultures were established from lung samples donated by 16 people with a doctor diagnosis of mild to moderate asthma (mean age 36 y and range 23–62 y) and 17 people without asthma (mean age 55 y and range 29–83 y). The lung samples were either bronchial biopsies or resected lung tissue obtained from people undergoing surgery for thoracic malignancies or lung transplantation. All samples were obtained with the donor's informed consent and approval from Sydney South West Area Health Service or Australian Red Cross. Approval for this study was granted by The University of Sydney Human Ethics Committee.

### 2.3. Airway Smooth Muscle Cell Culture

ASM bundles were dissected out from macroscopically normal lung samples and grown as explants. The cells from people who had no doctor diagnosis of asthma are referred to as nonasthmatic. ASM cells were maintained in culture as previously described [19] in Dulbecco's Modified Eagle's Medium (DMEM) (Sigma-Aldrich) supplemented with 10% v/v heat-inactivated foetal bovine serum (FBS), 100 units/mL penicillin G, 100 *μ*g/mL streptomycin sulphate, 25 *μ*g/mL amphotericin B, 4 mM L-glutamine, and 20 mM HEPES, at pH 7.4 (growth medium) and grown at 37°C in a humidified 5% CO_2_ in air atmosphere. The cells adopted a “hill and valley” growth pattern and were positive for *α*-smooth muscle actin and h-calponin using immunofluorescence [19, 20]. ASM cells harvested between passages 4 and 7 were used in experiments.

### 2.4. Histamine and Tryptase Effects on ASM Cell CXCL10 Production

To investigate the effects of histamine and tryptase on ASM cell CXCL10 production, ASM cells were seeded into the wells of 6- or 24-well plates at 1 × 10^4^ cells/cm^2^ in growth medium. After 7 days growth, the cells were serum-deprived for 48 h in FBS-free DMEM supplemented as described above and also with 0.1% v/v bovine serum albumin (BSA) (serum-free medium). The well cultures were left untreated or treated with histamine (1, 10, or 100 *μ*M), tryptase (0.2, 1.0, or 5.0 nM), or the vehicle for tryptase and heparin (50 *μ*M), as used previously [15]. Immediately afterwards the cytokines IL-1*β*, TNF-*α*, and IFN*γ*, alone and in combination (cytomix), were added to untreated and treated wells. The cytokines were each used at 10 ng/mL and all treatments were performed in duplicate.

To investigate which histamine receptor was involved in the effects of histamine on ASM cell CXCL10 release, the cells were treated in duplicate with either the H1 receptor antagonist chlorpheniramine at 1 *μ*M [15, 21] or the H2 receptor antagonist ranitidine at 50 *μ*M [15, 22], for 30 minutes prior to the addition of histamine followed by the cytokines.

To investigate whether the proteolytic activity of tryptase was involved in its effects on ASM cell CXCL10 release, an aliquot of the same tryptase batch was first heat-inactivated at 56°C for 30 minutes and then immediately added to the ASM cells, or the serine protease inhibitor leupeptin (50 *μ*M) [15] was added to tryptase-treated ASM cells 3 h after the addition of the cytokines.

After 4 hours cytokine stimulation, the cells in the well cultures were washed and total RNA was extracted using the guanidine thiocyanate/phenol chloroform method [23]. The RNA was reverse transcribed using RevertAid First Strand cDNA Synthesis Kit (Fermentas Life Sciences, Hanover, MD, USA) and the cDNA amplified by PCR using FAM-labeled human CXCL10 and VIC labeled 18srRNA TaqMan probes on an ABI Prism 7500 (Applied Biosystems) as described previously [18].

Alternatively, the ASM well cultures were incubated with the cytokines for 24 hours and then the culture medium (CM) was collected and stored at −20°C for later analysis of CXCL10 levels using the CXCL10 Duo-set ELISA kit and protocol (R&D Systems, Minneapolis, MN, USA).

### 2.5. Tryptase Effects on Recombinant Human CXCL10

To investigate whether tryptase can cleave CXCL10, recombinant human CXCL10 diluted in serum-free medium to 250, 500, and 1000 pg/mL, or the same volume of CM from cytokine-stimulated ASM cells, was added to a series of wells in microwell plates. Tryptase (1 nM), heat-inactivated tryptase, or tryptase followed by leupeptin (50 *μ*M) 3 hours later, was added to duplicate wells containing CXCL10 or the ASM cell CM and the plates were incubated at 37°C for 24 hours and then CXCL10 was detected using ELISA.

### 2.6. Human Lung Mast Cell Isolation and Activation

MC were isolated from macroscopically normal lung tissue dissected immediately by a pathologist from lung samples donated by 6 people undergoing surgery for thoracic malignancies. The MC were isolated within 24 hours of resection as previously described [14, 24]. Briefly, the lung parenchyma was chopped finely, washed, and digested with collagenase (7.5 mg/g tissue) and hyaluronidase (3.75 mg/g tissue) for 90 minutes at 37°C. MC in the cell suspension were positively selected using sheep anti-mouse IgG-coated magnetic beads (Dynal, Oslo, Norway), which were coated with a second anti-human CD117 (clone YB5B8; Bioscientific, Sydney, Australia) antibody (5 *μ*g/mL) just before use and resuspended at 1 × 10^6^ cells /mL in growth medium. The HLMC were activated with IgE (2.5 *μ*g/mL) and goat anti-human IgE (1 *μ*g/mL) (Calbiochem, CA, USA) immediately or left unstimulated. The HLMC culture supernatants (SN) were collected after 2 hours (MC 2 h SN) and fresh growth medium was added to the HLMC. This medium was collected 24 hours after the HLMC were first activated (MC 24 h SN). The MC 2 h and 24 h SN were stored at −20°C until immediately before use in the experiments described below.

### 2.7. ASM Cell Treatment with Human Lung Mast Cell Products

To investigate the effects of HLMC products released at different times after activation on ASM cell CXCL10 production, harvested ASM cells were placed in the wells of 96-well plates at a density of 3200 cells/100 uL/well in growth medium and incubated as described above. After 24 hours the cells were serum-deprived in serum-free medium for 72 hours. Then the ASM cells were stimulated with growth medium ± IFN*γ* (10 ng/mL) and, after 30 minutes, treated in triplicate or quadruplicate with the MC 2 h or 24 h SN (prepared as described above) at 0, 20, or 40% v/v in growth medium in the presence of the same concentration of IFN*γ*, or left untreated. This order was used to ensure that the ASM cells were activated by IFN*γ* as it induces CXCL10 production and the effects of the MC SN directly on its activity were not known. After 48 hours of treatment, the medium was collected from each ASM culture and stored at −20°C for later measurement of CXCL10 levels using ELISA. ASM cell CXCL10 release in response to growth medium ± IgE/anti IgE was also determined.

### 2.8. Protease Involvement in the Effects of HLMC Products Released by 2 h

To establish whether or not MC proteases were involved in modulating ASM CXCL10 release, 2 h MC SN or the same SN already pretreated for 30 minutes with 50 *μ*M leupeptin were added at 40% v/v in growth medium to the ASM cell well cultures. After 3 hours coincubation at 37°C, leupeptin was added to some of the wells containing untreated MC SN and the incubation was continued. All the culture medium was collected from each well after 48 hours and CXCL10 levels were quantified using ELISA.

To investigate if the effects of 24 h MC SN on cytokine-induced ASM CXCL10 could be due to TNF-*α* produced by MC, TNF-*α* levels were measured in the MC SN using ELISA. Then serum-deprived ASM cells were stimulated with IFN-*γ* (10 ng/mL), with and without a similar range of TNF-*α* concentrations to those detected in the MC SN, and made up in growth medium. After 24 h incubation at 37°C, the culture SN were collected and CXCL10 levels in them were quantified as above.

The effects of 2 h MC SN, *β*-tryptase, or leupeptin on the CXCL10 ELISA capture antibody were also investigated. Wells were coated with CXCL10 capture antibody and washed ready for use as usual; then either 40% v/v 2 h MC SN, 1 nM *β*-tryptase, 50 *μ*M leupeptin, or vehicle, in growth medium or growth medium alone, was added to the wells already containing capture antibody. After incubation together for 1 hour at 37°C, the wells were washed and doubling dilutions of CXCL10 standards were added and the remainder of the ELISA protocol was completed without modification.

### 2.9. Data Analysis

CXCL10 mRNA or protein levels from replicate treatments were averaged. The results for each experiment were expressed as a percentage of the cytokine control. The mean ± SEM was then calculated for the asthmatic and non-asthmatic cell lines and for each treatment. Statistical analyses were performed on all data using Statview (SAS Institute, Cary, North Carolina) or Prism 5.03 (GraphPad, La Jolla, CA) and significance (*P* < 0.05) was determined using 1-way or 2-way analysis of variance (ANOVA) with the Bonferroni correction for multiple comparisons or using the Student's *t*-test as appropriate.

## 3. Results

### 3.1. Histamine Effects on ASM Cell CXCL10 Production

In preliminary experiments we established that histamine did not induce ASM cell CXCL10 release. The levels of CXCL10 released by untreated ASM cells from people with and without asthma were very low (Table 1). Histamine treatment at 1, 10, or 100 *μ*M for 24 h did not alter CXCL10 release by the ASM cells (data not shown).

Similar to previous reports, cytokine stimulation increased ASM cell CXCL10 production. IL-1*β*, TNF-*α* and IFN*γ*, alone and in combination (cytomix), stimulated significant CXCL10 release from the asthmatic and non-asthmatic ASM cells (Table 1).

**Figure 1:**
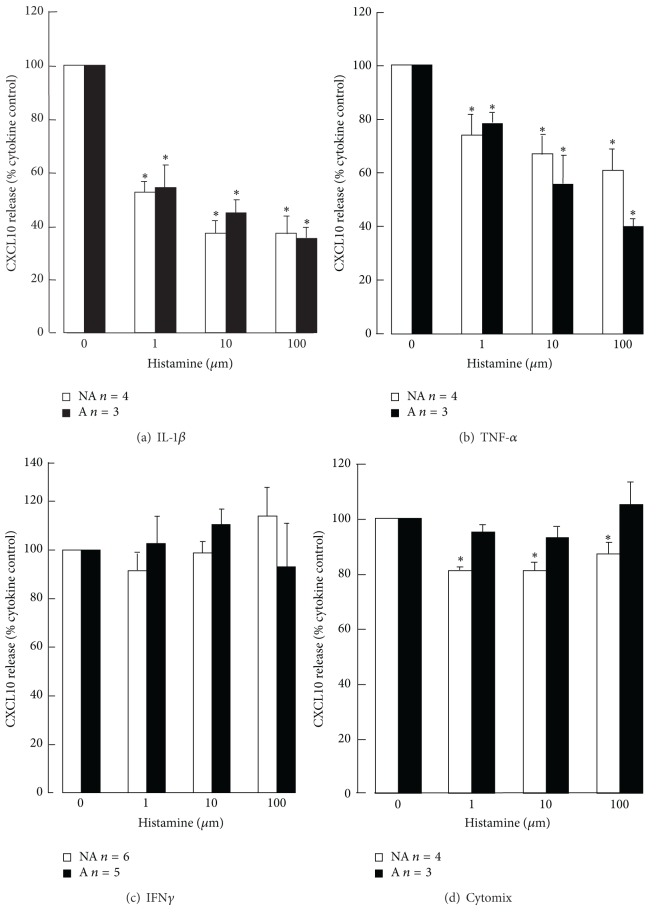
The effects of histamine on CXCL10 production by asthmatic (A) and nonasthmatic (NA) ASM cells. Confluent serum-deprived ASM cells were stimulated with the individual cytokines (a) IL-1*β*, (b) TNF*α*, (c) IFN*γ*, or (d) cytomix for 24 h and CXCL10 released into the culture medium quantified using ELISA. *P < 0.05 compared with the cytokine control, ANOVA.

Histamine had much greater inhibitory effects on IL-1*β*- and TNF-*α*-induced CXCL10 release than cytomix-induced release from both asthmatic and non-asthmatic ASM cells. The inhibitory effects of 1, 10, and 100 *μ*M histamine on IL-1*β*-, and TNF-*α*-induced CXCL10 were all significant and concentration-related (Figures 1(a) and 1(b)). Histamine at the highest concentration (100 *μ*M) reduced IL-1*β*-induced release from asthmatic (*n* = 3, *P* < 0.0001) and non-asthmatic (*n* = 4, *P* < 0.0001) ASM cells to a similar extent (35.7 ± 3.3% and 37.5 ± 6.5% of the IL-1*β* control, resp.) (Figure 1(a)). Whereas it reduced TNF-*α*-induced release from asthmatic (*n* = 3, *P* < 0.0001) and non-asthmatic (*n* = 4, *P* = 0.0013) ASM cells down to 39.6 ± 3.7% and 61.0 ± 7.9% of the TNF-*α* control, respectively, (Figure 1(b)), but this difference in effects on asthmatic and non-asthmatic cells did not achieve statistical significance. In contrast, histamine had no effect on IFN*γ*-induced CXCL10 release from asthmatic and non-asthmatic ASM cells (Figure 1(c)) but at each concentration did reduce cytomix-induced release from non-asthmatic cells very slightly (Figure 1(d)).

**Figure 2:**
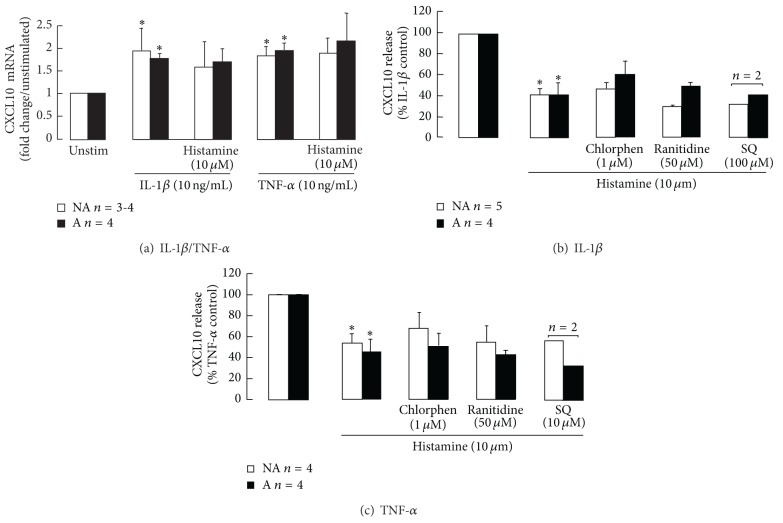
The effects of histamine on cytokine-induced (a) CXCL10 gene expression and (b) H1/H2 receptor involvement. Asthmatic (A) and nonasthmatic (NA) confluent serum-deprived ASM cells were left untreated or treated with the H1 or H2 receptor antagonists chlorpheniramine and ranitidine, respectively, for 45 minutes prior to and during stimulation with IL-1*β* or TNF-*α*. After 4 or 24 h stimulation cells were lysed and CXCL10 mRNA levels were measured using real-time RT PCR or CXCL10 in the culture medium quantified using ELISA, respectively. *P < 0.05 compared with (a) unstimulated cells (unstim) or (b) and (c) the cytokine control, ANOVA.

Although IL-1*β* and TNF-*α* both induced significant increases in CXCL10 gene expression over 4 hours, histamine 10 *μ*M did not affect CXCL10 mRNA levels induced by either cytokine (Figure 2(a)), even though it significantly reduced CXCL10 release. Further, the effects of 10 *μ*M histamine on CXCL10 release were not reversed by pretreating the asthmatic and non-asthmatic ASM cells with the H1 and H2 receptor antagonists chlorpheniramine and ranitidine, respectively, or with the cell membrane permeable adenylate cyclase inhibitor SQ 22,536 (Figures 2(b) and 2(c)).

### 3.2. Tryptase Effects on ASM Cell CXCL10 Production

Neither tryptase (0.2, 1, or 5 nM) nor the vehicle (heparin 50 *μ*M) induced CXCL10 release from ASM cells from people with and without asthma. The levels of CXCL10 were similar to those in the untreated controls (asthmatic: 0.15 ± 0.06 ng/mL, *n* = 3; non-asthmatic: 0.15 ± 0.07 ng/mL, *n* = 4).

Tryptase reduced the levels of CXCL10 detected in CM from asthmatic and non-asthmatic ASM cells. When tryptase (1 nM) was added to the ASM cells 30 minutes after the cytokines IL-1*β*, TNF-*α*, IFN*γ*, or cytomix, the CXCL10 levels detected in non-asthmatic and asthmatic ASM cell CM were very much lower than the respective cytokine control (Table 2). The heparin vehicle for tryptase did not affect cytokine-induced CXCL10 levels (data not shown).

**Figure 3:**
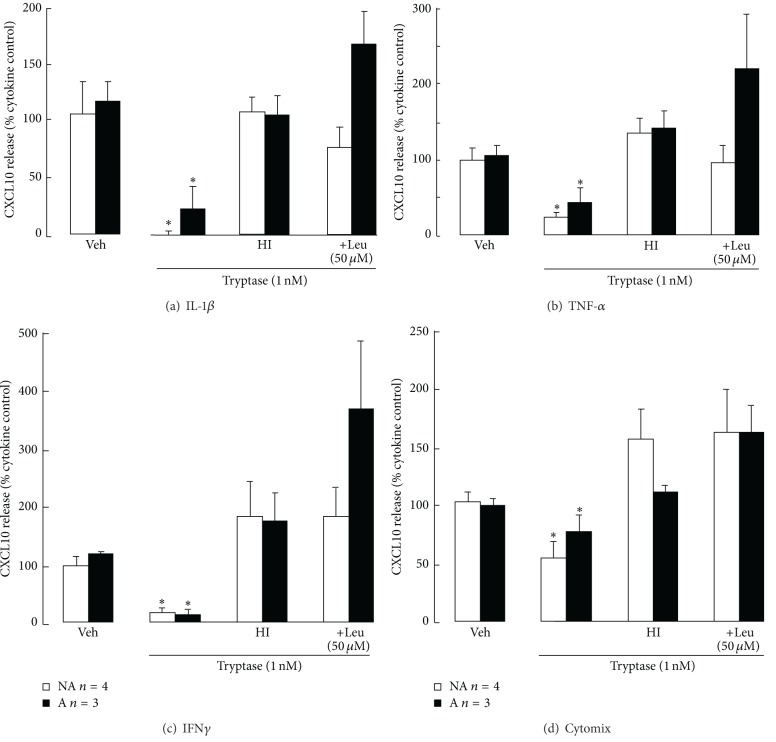
The proteolytic effects of tryptase on cytokine-induced CXCL10 production by asthmatic (A) and non-asthmatic (NA) ASM cells. Confluent serum-deprived ASM cells were stimulated with (a) IL-1*β*, (b) TNF*α*, (c) IFN*γ*, or (d) cytomixfor 30 minutes prior to and during treatment with tryptase, heat-inactivated tryptase (HI), or its vehicle (veh) for 24 h. Leupeptin (Leu) was added to some cell cultures 3 h after the cytokines. CXCL10 in the culture supernatants was detected using ELISA. *P < 0.05 compared with the vehicle control, ANOVA.

To investigate whether tryptase proteolytic activity was involved, the effects of tryptase and the same tryptase either heat-inactivated just before use or in the presence of the serine protease inhibitor leupeptin were compared. Untreated tryptase again markedly reduced nonasthmatic and asthmatic ASM cell cytokine-induced CXCL10 levels, whereas heat-inactivated tryptase did not affect them (Figures 3(a)–3(d)). Similarly, tryptase did not reduce CXCL10 levels when leupeptin (50 *μ*M) was added 3 hours after the cytokine(s). However asthmatic ASM cell CXCL10 levels were more variable in the presence of leupeptin. CXCL10 levels in CM from asthmatic IL-1*β*-, TNF-*α*-, or IFN*γ*-stimulated cells treated with tryptase followed by leupeptin were higher than levels in the presence of the same tryptase which had been heat-inactivated, but the differences were not significant (Figures 3(a)–3(d)).

### 3.3. Tryptase Effects on Recombinant Human CXCL10

**Figure 4:**
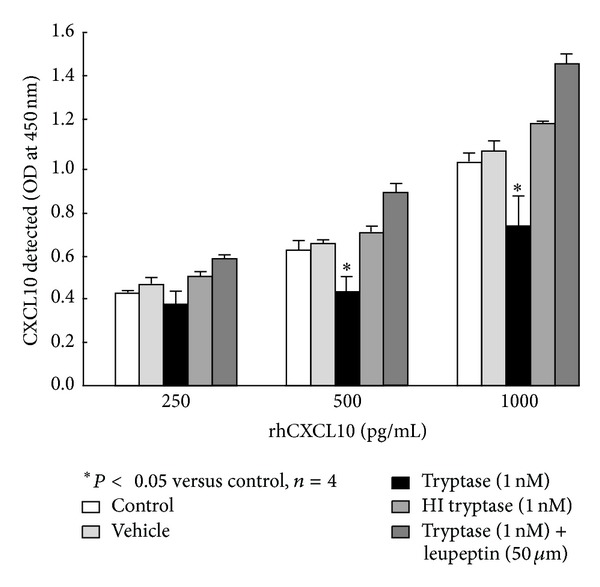
The proteolytic effects of tryptase on rhCXCL10 in a cell free system. The rhCXCL10 made up in serum-free DMEM supplemented with 0.1% BSA was placed in 96-well culture plates and incubated with tryptase ± leupeptin, heat-inactivated tryptase (HI), or its vehicle for 24 h under the same conditions as the ASM cells. CXCL10 was detected using ELISA. *P < 0.05 compared with the vehicle control, ANOVA.

The proteolytic effects of tryptase on CXCL10 were investigated further in a cell-free system. Significantly less CXCL10 was detected in wells containing human recombinant CXCL10 (500–1000 pg/mL in serum-free medium) following treatment for 24 hours at 37°C with 1 nM tryptase than with its vehicle (Figure 4). CXCL10 levels were not reduced in wells treated with heat-inactivated tryptase and were even increased in those treated with tryptase followed by leupeptin (50 *μ*M) 3 hours later. Similarly, tryptase treatment after-collection also markedly reduced detectable CXCL10 levels in culture medium collected from cytokine-stimulated ASM cells when compared to the vehicle-treated controls (data not shown). The capacity of the antibody from the Duo-set kit to capture CXCL10 standards was not affected by direct incubation with tryptase, its vehicle, or leupeptin at the concentrations used above (data not shown).

### 3.4. Effects of Human Lung Mast Cell Products on ASM Cell CXCL10

**Figure 5:**
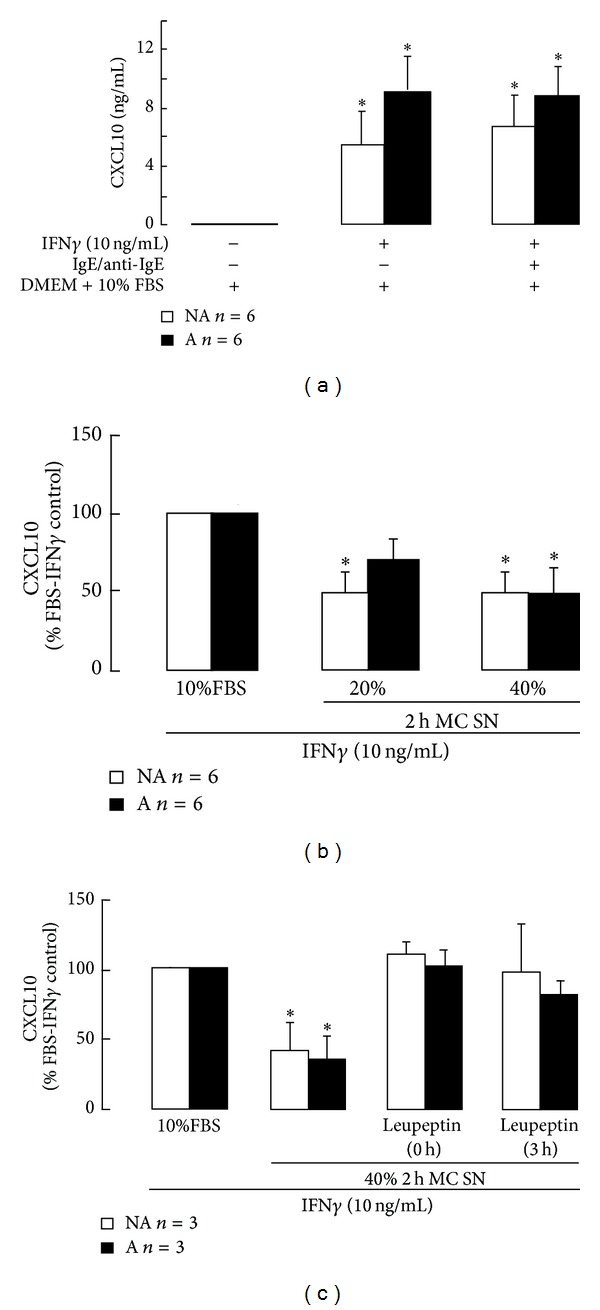
The effects of human lung mast cell products released early after activation on asthmatic (A) and non-asthmatic (NA) ASM cell CXCL10 production. Mast cells (MC) were isolated from human lung and immediately activated with IgE/anti-IgE in culture medium (DMEM + 10% FBS) and the culture supernatants collected after 2 hours (2 h MC SN). The MC SN were added at 20% and 40% v/v to ASM cells stimulated with IFN*γ* and CXCL10 levels were quantified after 48 hours stimulation using ELISA. ^∗^ *P* < 0.05 compared with the DMEM + 10% FBS control (a) or FBS-IFN*γ* control ((b) and (c)), ANOVA.

Asthmatic and non-asthmatic ASM cells responded in a similar way to the products released by human lung MC, but the effects of the 2 h and 24 h MC SN on ASM cell CXCL10 production differed. Although 10% FBS did not induce ASM cell CXCL10 release, IFN*γ* induced substantial release in its presence over the 48 hour incubation. CXCL10 release was generally greater from asthmatic ASM cells (9.3 ± 2.4 ng/mL, *n* = 6) than non-asthmatic ASM cells (5.4 ± 2.0 ng/mL, *n* = 6), but the difference was not statistically significant. ASM cell CXCL10 release was not affected by the presence of IgE/anti-IgE (Figure 5(a)).

The MC 2 h SN markedly reduced asthmatic and non-asthmatic ASM cell CXCL10 levels following IFN*γ* stimulation. At 20% v/v, the MC 2 h SN significantly reduced non-asthmatic ASM cell CXCL10 levels down to 47 ± 12% of the FBS-IFN*γ* control and at 40% v/v significantly reduced asthmatic ASM cell CXCL10 levels to 46 ± 15% of control (Figure 5(b)). To investigate whether this effect was due to MC protease activity affecting early events leading to CXCL10 production or affecting CXCL10 after new gene transcription had commenced, leupeptin (50 *μ*M) was added at two different steps. Leupeptin inhibited the effects of the MC 2 h SN irrespective of whether the SN were treated with it before they were added to the ASM cells (0 h), or it was added to the ASM cultures 3 hours after the SN (3 h) (Figure 5(c)). When the CXCL10 capture antibody was incubated with the MC 2 h SN there was no effect as detection of human recombinant CXCL10 in the ELISA remained the same as with the vehicle-treated and untreated capture antibody controls (data not shown).

**Figure 6:**
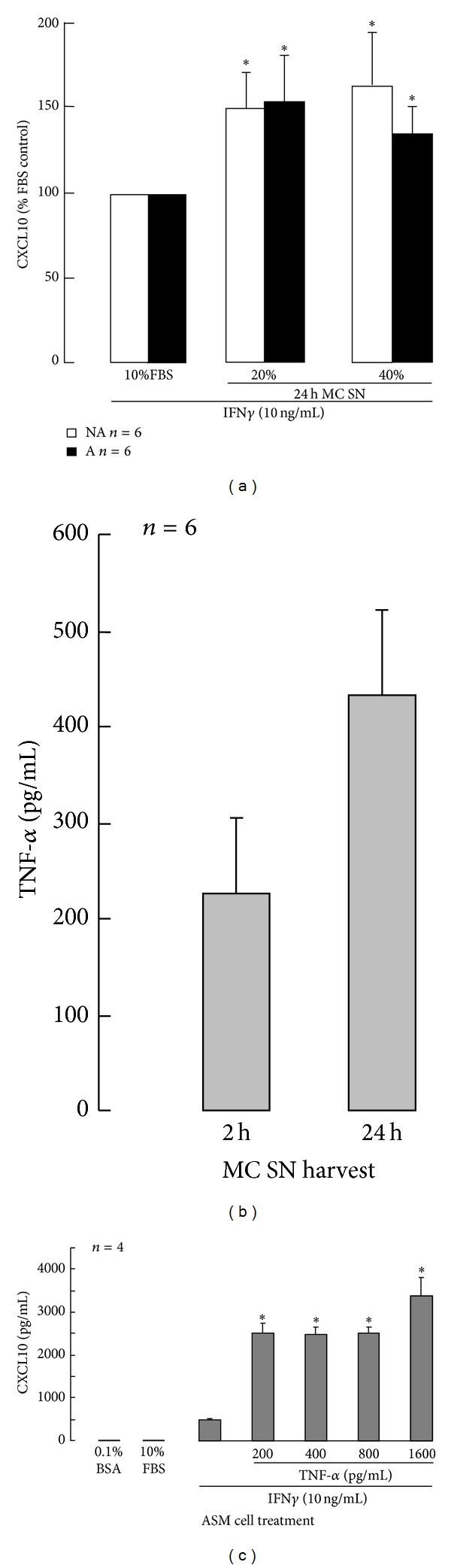
The effects of human lung mast cell products released later after activation (2–24 h) on asthmatic (A) and non-asthmatic (NA) ASM cell CXCL10 production. Mast cells (MC) were isolated from human lung and immediately activated with IgE/anti-IgE in culture medium (DMEM + 10% FBS). After 2 hours the culture supernatants were collected (2 h MC SN) and replaced by fresh medium. Further supernatants were collected at 24 hours (24 h MC SN). The 24 h MC SN were added at 20% and 40% v/v to IFN*γ*-stimulated ASM cells and after 48 hours CXCL10 levels were quantified using ELISA (a). TNF-*α* levels in the 2 h and 24 h MC SN were quantified using ELISA (b) and the effects of these low concentrations of TNF-*α* on IFN*γ*-induced CXCL10 production by ASM cells in DMEM + 10% FBS quantified (c). ^∗^ *P* < 0.05 compared with the DMEM + 10% FBS control ((a) and (c)), ANOVA.

In contrast, the MC 24 h SN increased asthmatic and non-asthmatic ASM cell IFN*γ*-induced CXCL10 release. The MC 24 h SN at 20% and 40% v/v significantly increased IFN*γ*-induced CXCL10 release from asthmatic cells up to 157 ± 28% and 137 ± 16% and from non-asthmatic cells up to 155 ± 22% and 170 ± 32% of the FBS-IFN*γ* control, respectively (Figure 6(a)). As TNF-*α* acts synergistically with IFN*γ* to increase CXCL10 production, the levels of TNF-*α* in MC SN were quantified. The MC 2 h and 24 h SN contained TNF-*α* at 227 ± 79 and 434 ± 87 pg/mL, respectively (Figure 6(b)). TNF-*α* over those concentrations increased ASM cell IFN*γ*-induced CXCL10 release by ≥5fold (Figure 6(c)).

## 4. Discussion

In asthma, the ASM produces the chemokine CXCL10 which we have previously shown induces MC chemotaxis through CXCR3 engagement [17]. CXCL10 may also mediate CXCR3^+^T lymphocyte recruitment to the ASM in severe asthma and following allergen challenge [25]. Thus CXCL10 may be critical in asthma because it can induce airway myositis. This is the first study to examine the effects of MC products on CXCL10 production by ASM cells from people with and without asthma. The effects of individual granule-derived MC products and all the products released by human lung MC in the first 2 or 2–24 hours following IgE receptor activation were investigated. The key granule-derived product histamine reduced ASM cell CXCL10 release induced by the proinflammatory cytokines IL-1*β* and TNF-*α*. As well, the proteolytic activity of tryptase, or the leupeptin-sensitive serine proteases released by human lung MC within two hours of activation, markedly reduced CXCL10 levels released following ASM cell cytokine stimulation. In contrast, human lung MC products synthesised and released later increased ASM CXCL10 release and levels of human lung MC-derived TNF-*α* were sufficient to amplify IFN*γ*-induced CXCL10 production. These findings are evidence that mast cells are able to regulate ASM cell CXCL10 production and degradation and thus may regulate their own and/or T cell recruitment to the ASM in asthma.

Following MC activation histamine, along with other products also stored in the granules, is released rapidly (reviewed in [10]). Histamine has a very broad spectrum of activities and histamine levels are high in the bronchoalveolar lavage fluid of people with asthma and correlate with airway hyperresponsiveness [26]. The contractile effects of histamine on ASM are well characterised and mediated via its H1 and H2 receptors, which are highly expressed on ASM. Histamine binding to the H1 receptor primarily activates phospholipase C-inositol triphosphate-diacylglycerol signalling and leads to Ca^2+^ release from intracellular stores and protein kinase C activation, both of which can also upregulate cAMP [27]. Signalling via H2 receptors activates adenylate cyclase to directly generate cyclic adenosine monophosphate (cAMP), but H2 receptors are also coupled to the phosphoinositide system [27]. Through the H1 receptor, histamine can also activate NF*κ*B [28, 29] and thus might be expected to enhance proinflammatory gene transcription. Histamine also modulates the activity of the coactivator cAMP response element-binding protein (CREB/CBP) to mediate some of its effects on gene expression [30, 31]. However in this study histamine reduced IL-1*β*- and TNF-*α*-induced CXCL10 release, but not gene expression, which is mediated via the transcription factor NF*κ*B [18, 32]. Neither the H1 nor the H2 receptor antagonists chlorpheniramine and ranitidine, respectively, prevented the inhibition of CXCL10 release.

Nevertheless, differences in transcriptional pathways following ASM cell stimulation with the individual cytokines and cytomix may contribute to the differential effects of histamine indirectly. In contrast to ASM CXCL10 production induced by IL-1*β* and TNF-*α*, IFN*γ*-induced production was not affected by histamine. Although Smith et al. [33] demonstrated that IFN*γ*, but not TNF-*α*, significantly increases histamine-induced inositol-phosphate signalling and H1R mRNA expression, those IFN*γ* effects do not match with the observations reported here. IFN*γ* activates the JAK_2_-STAT-1 pathway predominantly and NF*κ*B only weakly in non-asthmatic ASM cells to induce CXCL10 production [34]. As observed here and previously [18, 34], IFN*γ* synergistically increases CXCL10 production when used with IL-1*β* and/or TNF-*α*, but Clarke et al. [34] did not observe any effects on NF*κ*B or STAT-1 activation and DNA binding. Rather they found that the combined cytokines synergistically increase the recruitment of CREB binding protein (CBP) and RNA polymerase II to the CXCL10 promoter. Such IFN*γ*-induced increases in CREB binding to the CXCL10 promoter may overwhelm any inhibitory effects of histamine. Further, we have observed in asthmatic compared with non-asthmatic ASM cells that NF*κ*B activation is stronger and STAT-1 activation is weaker in response to the three cytokines combined (cytomix) [35], which may underlie the small inhibitory effect of histamine in the non-asthmatic ASM cells. Interestingly CXCL10 production induced by IFN*γ* or cytomix is also not inhibited by current asthma therapies such as fluticasone and/or salmeterol, whereas IL-1*β*- and TNF-*α*-induced release is markedly reduced by these agents [34, 36].

Histamine and cytokines may have opposing effects on intracellular (Ca^2+^) levels in human ASM cells. Mitochondria buffer cytosolic (Ca^2+^) levels [37] and histamine causes a rapid increase in both cytosolic and mitochondrial (Ca^2+^) levels. Whereas TNF-*α* decreases mitochondrial (Ca^2+^) levels through the mitochondrial Na^+^/Ca^2+^ exchanger, while increasing cytosolic (Ca^2+^) levels [37]. As indicated above, TNF-*α* also induces NF*κ*B mediated CXCL10 gene transcription [18, 32], whereas histamine does not. NF*κ*B mediated gene transcription is inhibited by SERCA pump inhibition [38], including CXCL10 production, especially in asthmatic ASM cells [35]. If histamine modulated TNF-*α*-induced changes in mitochondrial and cytosolic (Ca^2+^) levels to limit ASM cell CXCL10 production, reduced CXCL10 mRNA levels in histamine-treated cells would have been observed in this study but they were not.

The lack of effect of the H1 and H2 receptor antagonists was surprising in view of our previous findings. Firstly, chlorpheniramine prevented histamine-induced changes in ASM cell GM-CSF and RANTES release following stimulation by IL-1*β* or TNF-*α* [15]. Secondly, the long acting *β*_2_-adrenoceptor agonist salmeterol which, like histamine, increases cAMP levels also inhibited ASM cell CXCL10 release induced by these cytokines [36]. Further, the effects of histamine appeared to be posttranscriptional, as cytokine-induced CXCL10 gene expression was not affected. Posttranscriptional effects of histamine on keratinocytes have been reported but were mediated via the H1 receptor [39]. To our knowledge, H3 receptors are expressed on cholinergic nerves, but not ASM in the airways [40]. Gantner and colleagues [41] used specific primers for receptors H1-4 and detected mRNA expression for H1, H2, and H4 receptors, but not for H3 receptors. Perhaps the indirect inhibitory effects of histamine observed in this study were mediated via the Gi/o coupled H4 receptors. H4 receptors play an important role in the regulation of immune responses, including chemokine production, in the skin [42] and so might also regulate ASM cell chemokine production. Further studies of cytokine-stimulated ASM cell CXL10 production in the presence of histamine are needed to determine the molecular mechanisms underlying its effects and histamine receptor involvement. Recently developed selective agonists (e.g., 4-methylhistamine) and antagonists (e.g., JNJ7777120) for H4 receptors would be useful to clarify its role [43].

ASM cells also express the organic cation transporter 3 (OCT3 or EMT) in addition to histamine receptors [44]. Histamine is a good substrate for OCT3 [45] and so it is possible that histamine entered the ASM cells stimulated with IL-1*β* and TNF-*α* via OCT 3 and affected posttranscriptional events involved in CXCL10 protein synthesis/release. Further studies are needed to investigate the above possibilities and the mechanisms involved in any effects of histamine.

Importantly, this study is the first demonstration that CXCL10 is susceptible to digestion by serine proteases such as tryptase. The levels of CXCL10 released following cytokine stimulation were reduced by tryptase, which is stored in the granules as a tetrameric complex with proteoglycans such as heparin and released at the same time as histamine. Unlike histamine, tryptase reduced detectable CXCL10 levels irrespective of the individual cytokine used to stimulate the ASM cells. This effect was due to tryptase protease activity as it was prevented by heat-inactivation and the serine protease inhibitor leupeptin. Further it was likely due, at least in part, to direct tryptase proteolysis of CXCL10, as tryptase reduced the levels of CXCL10 detected in a cell-free system. In addition, leupeptin added 3 h after any of the cytokines, when there is already a substantial increase in CXCL10 mRNA expression [18], completely prevented the reduction in cytokine-induced CXCL10 levels. These findings add CXCL10 to the chemokines CCL11 (eotaxin) and CCL5 (RANTES) that are also produced by ASM cells and cleaved by tryptase *in vitro *[13] or by ASM cell cocullture with MC [46].

Although other studies have investigated the effect of MC products individually on ASM cell synthetic function as above, this is the first study to also examine the overall effects of MC products released at different times after MC activation on asthmatic and non-asthmatic ASM cell CXCL10 production. As ASM cells do not release CXCL10 constitutively, or in response to FBS, IFN*γ* was used to induce CXCL10 release so the effects of the MC products on it could be assessed. The effects of rapidly released human lung MC products (2 h SN) on ASM cell CXCL10 levels complement the effects obtained with tryptase. Early MC products reduced asthmatic and non-asthmatic ASM cell IFN*γ*-induced CXCL10 levels by a similar amount. These effects were also prevented by leupeptin added later, indicating that serine proteases were involved, but do not affect early CXCL10 gene transcription.

As well, a role for histamine cannot be completely dismissed. MC supernatants generated in the manner used here contain significant amounts of histamine [47], which would have been ineffective against the IFN*γ*-induced CXCL10 production used in this study. Early release of histamine may limit CXCL10 production generated under different inflammatory conditions when IFN*γ* is absent.

In contrast to the effects of the MC products released early, newly synthesised products released sometime later (24 h SN) increased IFN*γ*-induced CXCL10 levels. Again the proportional effects of the MC products on ASM cells from people with and without asthma were similar. MC produce TNF-*α* [48], which synergistically increases IFN*γ*-induced CXCL10 production by ASM cells *in vitro* [18, 32, 34]. This study has extended those findings by providing evidence that products released from human lung MC activated by IgE also increase CXCL10 release and include sufficient TNF-*α* to cause such an increase. Although TNF-*α* levels in MC SN collected early were half those in the SN collected later, they were still sufficient to amplify IFN*γ*-induced CXCL10 release. However no increase was observed, probably because the granule-derived proteases present in the SN cleaved the CXCL10 after it was released and thus reduced the amount of protein detected.

In this study, the effects of MC products released together, either early or later, after IgE receptor activation were examined *in vitro* on ASM cells obtained from people with and without asthma. The MC could only be isolated from larger lung samples as they became available and were all from people without asthma. The effects of MC from people with asthma on asthmatic ASM cell CXCL10 production may be different. However different percentages of the MC SN were used to reflect what occurs *in vivo*, where activated MC are present in the ASM layer in different numbers in asthma [4, 5, 7]. Thus not all ASM cells are in direct contact with a MC, but they have been observed in close proximity to each other [6, 7]. Given this proximity, it is apparent from our study that MC products could still affect ASM chemokine production in the absence of cell-cell contact.

## 5. Conclusions

This is the first study to examine the effects of MC products on CXCL10 production by ASM cells. Activated MC numbers are increased in the ASM in eosinophilic asthma and T-lymphocytes are present in the ASM in severe asthma and following allergen challenge. ASM-derived CXCL10 production may play a role in the recruitment of these cells to the ASM as both cell types express the CXCL10 receptor receptor CXCR3. In this study we have demonstrated that MC products released from granules soon after cell activation, or newly synthesized and then released later, differentially modulated levels of CXCL10 released by ASM cells from donors with and without asthma. Thus mast cells in the ASM have the potential to up- and downregulate chemokine levels locally and thereby affect their own recruitment and that of other inflammatory cells to the ASM in asthma.

## Figures and Tables

**Table 1:** Cytokine-induced CXCL10 release by ASM cells.

Cytokine	CXCL10 Release (ng/mL, mean ± SEM)	
Treatment	Asthmatic (*n* = 5)^1^	Non-asthmatic (*n* = 5)
Unstimulated	0.16 ± 0.04 (*n* = 6)	0.10 ± 0.03 (*n* = 7)
IL-1*β*	1.34 ± 0.27*	7.35 ± 3.53*
TNF-*α*	6.45 ± 1.56*	20.71 ± 9.44*
IFN*γ*	17.06 ± 11.57* (*n* = 6)	13.27 ± 10.0* (*n* = 7)
Cytomix	49.57 ± 16.21**	66.56 ± 14.44**

^1^Except where indicated; *P ≤ 0.01 and **P < 0.001 compared to unstimulated, paired *t*-test.

**Table 2:** The effects of tryptase on cytokine-induced CXCL10 release.

Cytokine	CXCL10 Release (% cytokine control, mean ± SEM)	
Stimulus	Asthmatic (*n* = 4)	Non-asthmatic (*n* = 7-8)
IL-1*β*	22.8 ± 14.5*	9.0 ± 3.6*
TNF-*α*	36.3 ± 14.4*	25.2 ± 5.1*
IFN*γ*	11.1 ± 4.3*	13.6 ± 3.5*
Cytomix	65.8 ± 15.8*	42.8 ± 9.4*

*P < 0.01 compared to cytokine control, paired *t*-test.
